# Trends in kidney transplantation and living donor nephrectomy in Germany: a total population analysis from 2006 to 2021

**DOI:** 10.1007/s00345-023-04737-w

**Published:** 2024-01-10

**Authors:** Aristeidis Zacharis, Philipp Reimold, Cem Aksoy, Jonas Jung, Thomas Martin, Nicole Eisenmenger, Smita George Thoduka, Christer Groeben, Johannes Huber, Luka Flegar

**Affiliations:** 1 Department of Urology, Philipps University of Marburg, Baldingerstraße, 35043 Marburg, Germany; 2 Reimbursement Institute, Hürth, Germany; 3 Department of Nuclear Medicine, Philipps University of Marburg, Marburg, Germany

**Keywords:** Kidney transplantation, Surgical approach, Health services research, Robotic surgery, Laparoscopy

## Abstract

**Purpose:**

To analyze recent trends of surgical access routes, length of hospital stay (LOS), and mortality in kidney transplantation (KT) and living donor nephrectomy (LDN) in Germany.

**Materials and methods:**

We studied the nationwide German hospital billing database and the German hospital quality reports from 2006 to 2021.

**Results:**

There were a total of 35.898 KTs. In total, 9044 (25%) were living donor transplantations, while 26.854 (75%) were transplantations after donation after brain death (DBD). The share of open LDN decreased from 82% in 2006 to 22% in 2020 (− 4%/year; *p* < 0.001). The share of laparoscopic LDN increased from 18% in 2006 to 70% in 2020 (+ 3%/year; *p* < 0.001). The share of robotic LDN increased from 0% in 2006 to 8% in 2020 (+ 0.6%/year; *p* < 0.001). Robotic-assisted KT increased from 5 cases in 2016 to 13 procedures in 2019 (*p* = 0.2). LOS was shorter after living donor KT, i.e., 18 ± 12.1 days versus 21 ± 19.6 days for DBD renal transplantation (*p* < 0.001). Moreover, LOS differed for open versus laparoscopic versus robotic LDN (9 ± 3.1 vs. 8 ± 2.9 vs. 6 ± 2.6; *p* = 0.031). The overall in-hospital mortality was 0.16% (*n* = 5) after LDN, 0.47% (*n* = 42) after living donor KT and 1.8% (*n* = 475) after DBD KT.

**Conclusions:**

There is an increasing trend toward minimal-invasive LDN in recent years. Overall, in-hospital mortality was low after KT. However, 5 deceased healthy donors after LKD caution that the risks of this procedure should also be taken very seriously.

**Supplementary Information:**

The online version contains supplementary material available at 10.1007/s00345-023-04737-w.

## Introduction

Kidney transplantation (KT) presents the most effective treatment in patients with end-stage renal disease (ESRD) [1]. Remarkable progress in surgical techniques as well as immunosuppressive strategies has resulted in a substantial improvement of short- and medium-term outcomes for KT in recent years [2, 3]. In addition, KT improves patient survival, quality of life and has been shown to be more cost-effective than dialysis treatment [2, 4].

As the demand for kidney transplants rises and the availability of organs from donations after brain death (DBD) falls short, the significance of living kidney donation continues to grow each year. This trend is driven by the increasing number of patients with ESRD awaiting a suitable kidney transplant. Classically, KT as well as living donor nephrectomy (LDN) was performed as an open surgical procedure [5]. In the past years, significant progress in laparoscopic surgical techniques as a minimally invasive approach has extended their applications to urology and transplantation medicine, particularly in LDN [6, 7]. Recently, robotic KT has been introduced in select centers across Europe and globally [8, 9]. In general, these minimal invasive procedures are related to shorter length of hospital stay (LOS) as well as decreased mortality [10]. However, population-based data on KT surgical trends are scarce.

Therefore, the aim of the present study was to provide a current overview of surgical approach, LOS and in-hospital mortality for LDN and KT in Germany from 2006 to 2021.

### Patients and methods

In this study, we performed an analysis using data from German hospitals' quality reports and the German Federal Statistical Office (Destatis). The hospitals' quality reports were employed to identify national providers, while the Destatis database was used for analyzing all surgical procedures (Suppl. Table 1). We previously described the methods of data extraction and cohort identification [11].

#### German Federal Statistical Office (Destatis)

In 2004, the German healthcare system implemented international Diagnosis Related Groups (DRG) to regulate the reimbursement of inpatient treatments. These DRGs consist of diagnosis codes using the ICD-10 (International Statistical Classification of Diseases and Related Health Problems) and an OPS code (German adaptation of the International Classification of Procedures in Medicine) for the performed interventions. The data for each treated case are initially transferred to the German Federal Statistical Office (Destatis). The nationwide Destatis database encompasses every reimbursed inpatient case in Germany, except for cases from psychiatric, forensic, and military hospitals.

For our analysis, we used the OPS code “5-555.1” for DBD renal transplantation and “5-555.0” for living donor transplantation from 2006 to 2020. Further we analyzed the OPS codes “5-554.80” and “5-554.81” (open LDN), “5-554.83” (laparoscopic LDN) and “5-554.8” (LDN) in combination with the code “5-987” (robotic approach). Further we analyzed LOS and in-hospital mortality.

#### Quality reports

Starting from 2005, German hospitals have been under a legal obligation to furnish information about their work and structures through quality reports. We used the analysis tool “reimbursement.INFO” (RI Innovation GmbH, Hürth, Germany) to extract data on hospitals performing KT as well as LDN for the years 2006 to 2021. Due to data protection reasons, for small annual caseloads of 1, 2, or 3 cases, the exact number was not disclosed and instead reported as 1.

Maps were generated using the software “EasyMap 11.1 Standard Edition” (Lutum + Tappert DV-Beratung GmbH, Bonn, Germany).

#### Statistical analysis

The data were presented using absolute and relative frequencies. We used Chi-square and ANOVA test for group comparisons. To identify trends over time, linear regression models were utilized. Statistical significance was defined as *p* < 0.05. The statistical analysis was conducted using SPSS 28.0.1.1. (IBM Corp., Armonk, NY, USA).

#### Ethics statement

The data presented in this study were collected in compliance with the latest version of the World Health Organization Declaration of Helsinki. Since the data extracted from databases were appropriately anonymized and de-identified before being released, obtaining patient informed consent was not necessary, and no additional ethics statement was required for our study.

## Results

A total of 35.898 KT and 9.141 LDN were analyzed between 2006 and 2020 in Germany. In total, 9.044 (25%) were living donor KT, while 26.854 (75%) were DBD KT. In 2021, 69% of patients undergoing KT were older than 45 years of age. Further, the share of female patients receiving living donor KT in 2021was 34%, while the share for DBD KT was 36%, respectively.

**Fig. 1:**
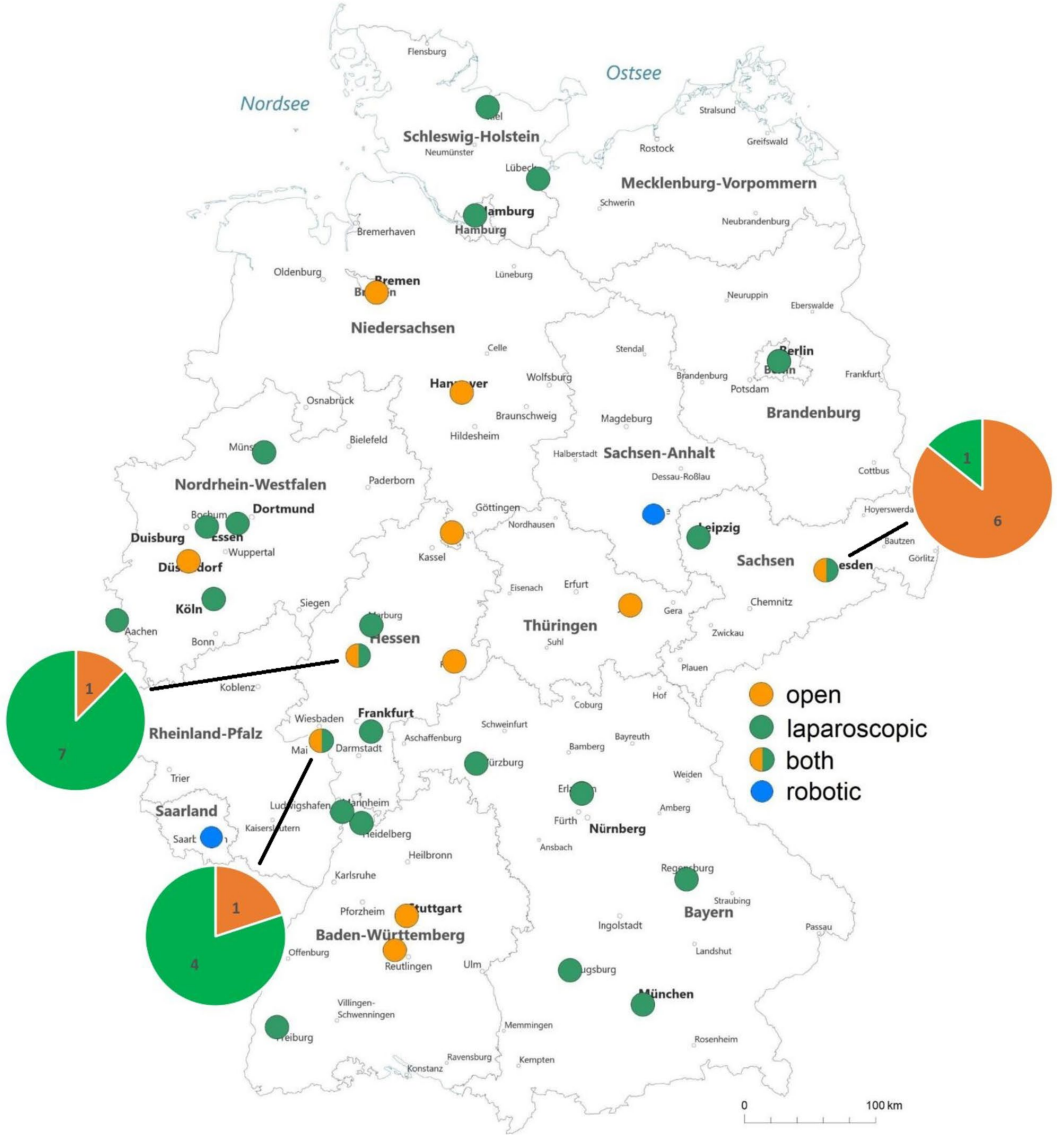
Geographical distribution for LDN according to surgical approach in 2021 (source: Quality reports, adopted according to center data from University Hospitals Halle and Homburg/Saar)

In 2021, 20 German centers performed exclusively laparoscopic LDN, while 8 centers performed exclusively open LDN and 3 centers offered both approaches (Fig. 1 and Suppl. Table 2). For laparoscopic LDN 9 centers performed < 5 cases, 8 centers performed 6–10 cases, and 12 centers performed > 10 cases. For open LDN 6 centers performed < 5 cases, 3 centers performed 6–10 cases, and 2 centers performed > 10 cases (*p* = 0.3; Suppl. Table 3).

**Fig. 2:**
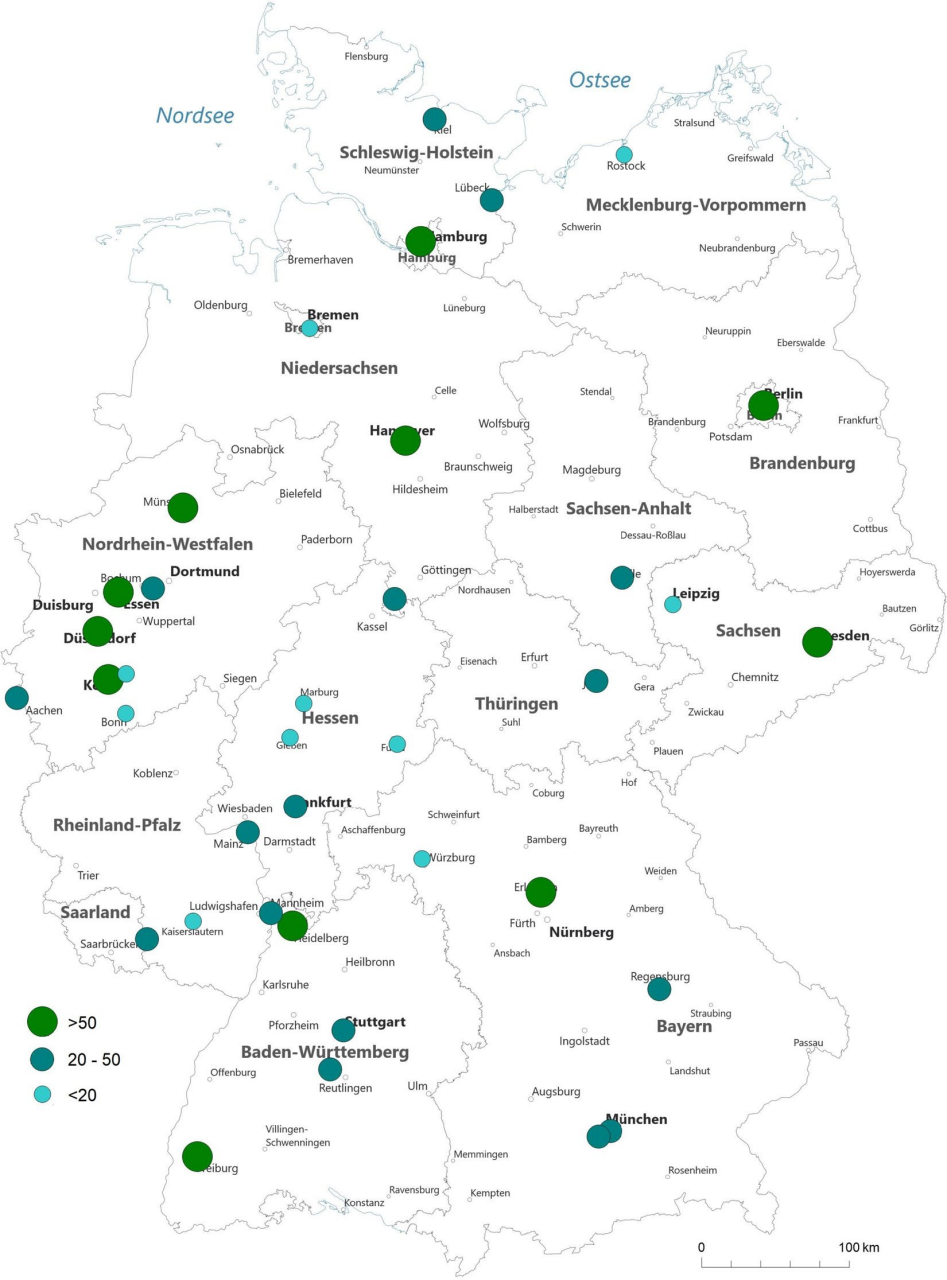
Geographical distribution of transplant centers performing KT in 2021 according to yearly caseload (source: Quality reports)

In 2021, 10 transplant centers performed 0–25 KTs, 16 transplant centers performed 25 to 50 KTs, and 11 transplant centers performed over 50 KTs per year (Fig. 2 and Suppl. Table 2).

### Surgical approach

**Fig. 3:**
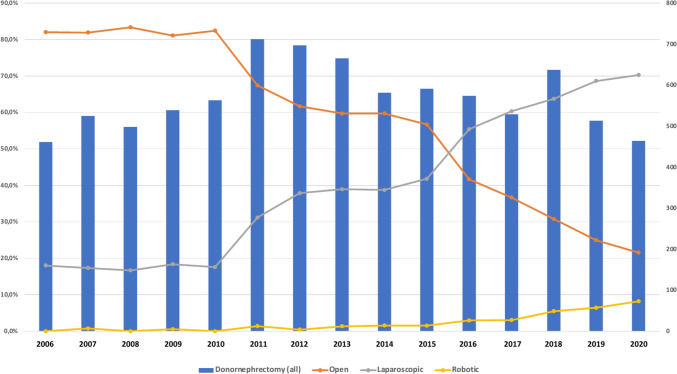
Surgical approach for LDN from 2006 to 2020. The blue columns represent the total caseload per year. The lines indicate the share of open (orange), laparoscopic (gray) and robotic approaches (yellow) (*Source*: Destatis)

The share of open LDN decreased from 82% in 2006 to 22% in 2020 (− 4%/year; *p* < 0.001). The share of laparoscopic LDN increased from 18% in 2006 to 70% in 2020 (+ 3%/year; p < 0.001). The share of robotic LDN increased from 0% in 2006 to 8% in 2020 (+ 0.6%/year; *p* < 0.001) (Fig. 3).

Robotic-assisted KT increased from 5 cases in 2016 to 13 procedures in 2019 (*p* = 0.2).

### Length of stay (LOS)

**Fig. 4:**
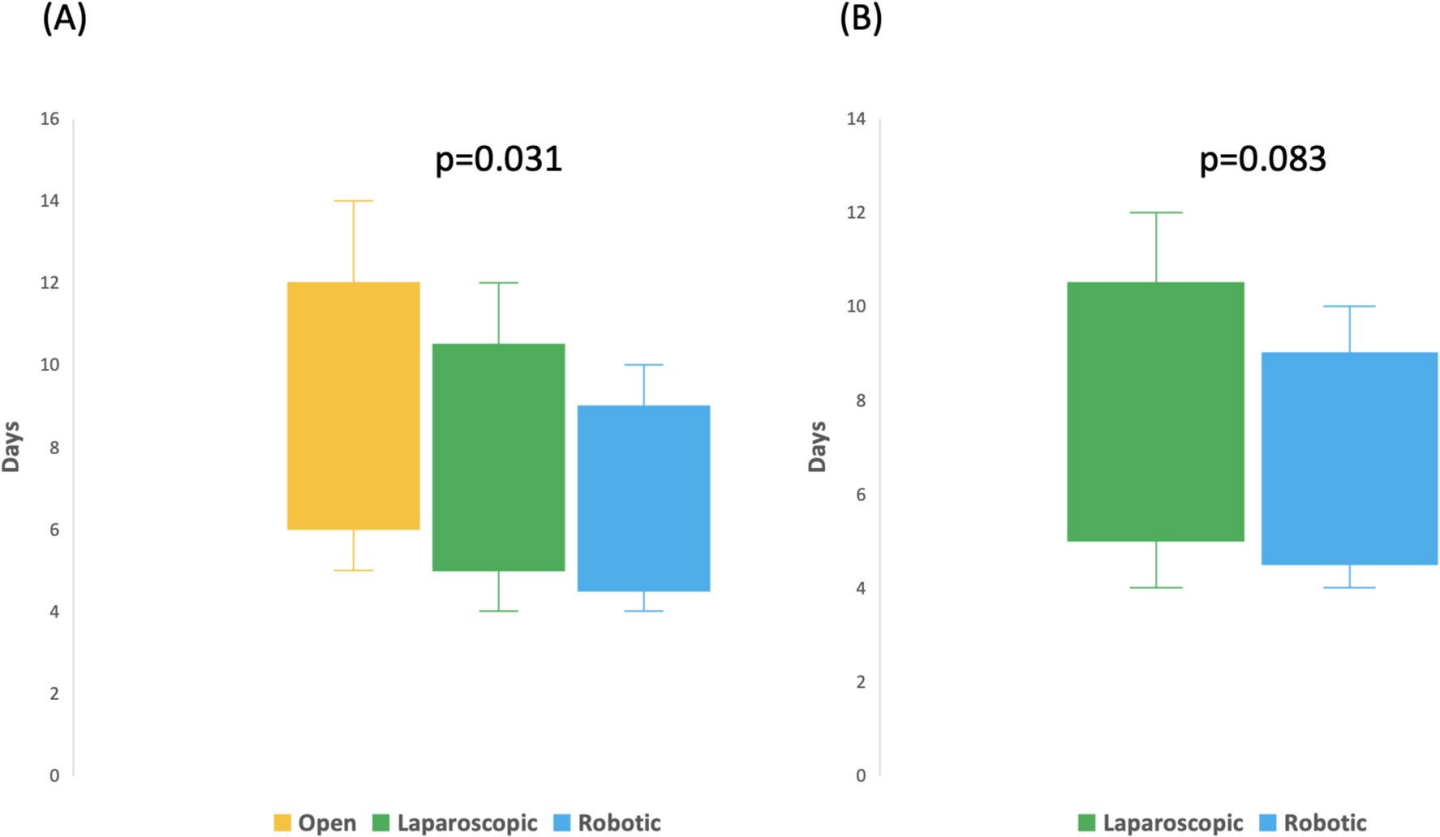
LOS after open versus laparoscopic versus robotic LDN (**A**) and after laparoscopic versus robotic LDN (**B**) (*Source*: Destatis)

LOS differed for open versus laparoscopic versus robotic LDN (9 ± 3.1 versus 8 ± 2.9 versus 6 ± 2.6; *p* = 0.031) (Fig. 4A). No statistical significance in LOS was proven between robotic and laparoscopic LDN; *p* = 0.083 (Fig. 4B). The overall LOS was shorter after living donor KT, i.e.,18 ± 12.1 days versus 21 ± 19.6 days for DBD KT (*p* < 0.001). Moreover, LOS decreased for living donor KT from 20 ± 13.1 days in 2006 to 17 ± 13.4 days (*p* < 0.001) in 2020, while for DBD KT it decreased from 23 ± 19.1 days in 2006 to 19 ± 18.9 days in 2020.

### Hospital mortality

The overall in-hospital mortality was 0.16% (*n* = 5) after LDN between 2006 and 2020. The overall in-hospital mortality was 0.47% (*n* = 42) after living donor KT and 1.8% (*n* = 475) after DBD KT.

## Discussion

In our population-based analysis of surgical trends for KT and LDN in Germany between 2006 and 2021, we made several important observations. In brief, we found a notable establishment of minimally invasive LDN, particularly in centers with high expertise. Additionally, we observed a low but significant mortality rate and identified an "optimization potential" in the regional distribution of KT centers.

Our analysis showed that the share of open LDN decreased significantly from 82% in 2006 to 22% in 2020. In the same period, the share of laparoscopic LDN increased from 18 to 70%, while the share of robotic LDN increased to 8% in 2020. These results are in line with a recently published analysis by Mankiev et al., where 71% of all transplant centers in Turkey used minimally invasive techniques in 2019 [12]. In general, laparoscopic LDN is despite the steep learning curve associated with faster recovery, less pain, less blood loss, earlier return to work, and better quality of life as compared with the conventional open approach [5, 12, 13]. Another important aspect to consider is that declining numbers of DBD has led to an even greater organ shortage in Germany in recent years. Furthermore, unlike other European countries, Germany does not currently implement the concept of donation after circulatory death (DCD), which aims to enhance organ donation rates [14]. Consequently, there has been a notable increase in the willingness of the population to consider living kidney donation as an option. This surge in interest has been especially supported by significant advancements in minimally invasive surgical techniques, such as laparoscopic and robotic-assisted surgery, which have extended their applications to transplantation medicine [15, 16].

Upon further analysis of our created map, it was observed that throughout Germany, 20 transplantation centers offered laparoscopic LDN, while only 8 centers performed classic open donor nephrectomy. Additionally, in 2021, robotic LDN was performed at only 2 German transplant centers. Numerous studies have demonstrated excellent perioperative and short-term outcomes for robotic LDN, indicating that the procedure is both safe and efficient [17, 18]. In 2001, the first series of robotic-assisted laparoscopic donor nephrectomies were reported [19]. Since then, the number of robot-assisted nephrectomies has been steadily increasing. A meta-analysis investigating 41 studies with over 32.000 minimally invasive LDN from 2016 showed that robotic LDN accounted for approximately 1.3% [20].

Additionally, our data showed that 13 robotic KTs were performed in 2019 in Germany. Robotic KT was first purely performed in 2010 by Giulianotti and colleagues [21]. The robotic approach in KT represents an advanced procedure that requires a high level of expertise. The potential advantages of RAKT include superior vascular anastomosis quality, a low complication rate, minimal postoperative pain, and rapid recipient recovery [22]. Recently, first studies have reported promising results for robotic KT from post-mortal donors [23]. However, a time-efficient organization of the transplantation pathway has to be considered.

Second, we observed that the overall LOS was shorter after living donor KT compared to DBD KT. This difference is mainly attributed to careful patient selection [17]. Further, LOS decreased significantly for living donor renal transplantation as well as for DBD transplantation from 2006 to 2020. In general, early discharge after surgical procedures has been lately proposed to reduce healthcare expenditures. A recent study showed that early discharge after KT appears to be cost-efficient and not associated with inferior post-transplant survival or increased readmission at 90 days [24]. Our analysis showed further that LOS differed significantly for open versus laparoscopic versus robotic nephrectomy for LDN (9 ± 3.1 vs. 8 ± 2.9 vs. 6 ± 2.6; *p* = 0.031). Several studies showed similar results with minimal-invasive LDN having shorter LOS compared to open LDN [10, 25]. Windisch et al. compared laparoscopic and robotic living donor nephrectomy and were able to show that RDN had a shorter hospital stay [18]. Our data showed no statistical significance between laparoscopic and robotic LOS.

Third, in the present study the overall in-hospital mortality was 0.16% after LDN, while the overall in-hospital mortality was 0.47% after living donor transplantation and 1.8% after DBD renal transplantation. Goyal and colleagues reported a perioperative mortality of 0.5% for KT between 2004 and 2013 from the National Inpatient Sample for the USA [26]. A Korean study from 2020 by Kim et al. described a treatment-related mortality of 1.7% and 4.1% within 1 and 3 months after KT. The authors identified old age, particularly greater than 70 years, donor status, and a high glucose level prior to KT were common risk factors for treatment-related mortality [27]. Further, a systematic review from 2022 providing an overview of different surgical techniques for LDN showed no mortality among kidney donors [28]. However, 5 deceased healthy donors after LKD in Germany within 15 years caution that the risks of this procedure should still be taken very seriously.

Finally, the rising popularity of robotic LDN and robotic KT presents a promising opportunity to address the ongoing decline in DBD renal transplantations. Urologists, given their extensive familiarity with robotic surgery, can play a pioneering role in advancing these techniques and driving their adoption to enhance KT outcomes [29].

We present comprehensive data on the trends, mortality, LOS and surgical treatment approaches concerning KT and LDN in Germany. However, a few shortcomings must be addressed. The quality reports lack clinical data such as underlying disease or comorbidities. Further, the quality of population-based data is always inferior to case files as well as study records and may be subject to documentation errors since they are prepared by the hospitals during routine care [30]. Moreover, we cannot link mortality to number of donations or surgical access routes. A correlation of hospitalization time with the number of performed procedures was also not possible to perform. However, for certain questions this data source provides high accuracy. For example, LOS and in-house mortality with regard to this total population sample are extremely precise outcomes.

To conclude, an increasing trend toward minimal invasive LDN was observed in recent years. Overall, in-hospital mortality was low for KT and LOS significantly shorter in robotic and laparoscopic living kidney donation. Robotic KT has only been performed at two urologic transplant centers but has the potential due to its good outcomes, to further help promoting living kidney donation as well as increasing transplantation numbers.

## Supplementary Information

Below is the link to the electronic supplementary material.

Supplementary file1 (DOCX 18 kb)

## Data Availability

All datasets used in this work are stored centrally at the specific institutes (German Federal Statistical Office—Destatis; German National Centre for Cancer Registry). The quality reports are online openly available.

## Abbreviations

DCD: Donation after circulatory death
DBD: Donation after brain death
KT: Kidney transplantation
LDN: Living donor nephrectomy
