# Supplementary material for: Trends in kidney transplantation and living donor nephrectomy in Germany: a total population analysis from 2006 to 2021
Source: World J Urol. 2024 Jan 10;42(1):24. doi: 10.1007/s00345-023-04737-w (PMC10781803; doi:10.1007/s00345-023-04737-w)
Supplement: Supplementary file 1 — Supplementary file1 (DOCX 18 kb) [file 345_2023_4737_MOESM1_ESM.docx]

**Supplemental Material**

**Suppl. Table 1.** Overview of the queried databases.

| **Data source** | Nationwide hospital billing database of the German Federal Statistical Office  (Destatis database) | German hospitals’ quality reports  (reimbursement.INFO tool) |
| --- | --- | --- |
| **Data details** | - Age and gender - Diagnosis code - Type of surgery and approach - Hospital characteristics (teaching status, size, annual surgery caseload, approaches for surgery) | - Age and gender - Type of surgery - Hospital characteristics (teaching status, annual surgery caseload) - Geographical localization of respective hospitals |
| **Data query option** | - Combination of OPS- and DRG code possible | - Only OPS- or DRG code |
| **Number of patients** | 35.898 | 23.285 |
| **Proportion of the country** | 100% | 100% |
| **Included years** | 2006 - 2020 | 2006 – 2021 (years 2007, 2009 and 2011 missing) |

**Suppl. Table 2.** Transplantation centers performing LDN and KT in 2021.

| **n** | **Centers LDN** | **Transplantation Centers** |
| --- | --- | --- |
| 1 | Köln | Berlin |
| 2 | Berlin | Hannover |
| 3 | Heidelberg | Essen |
| 4 | Münster | Düsseldorf |
| 5 | Freiburg | Münster |
| 6 | München | Heidelberg |
| 7 | Hamburg | Dresden |
| 8 | Essen | Freiburg |
| 9 | Düsseldorf | Hamburg |
| 10 | Halle (Saale) | Erlangen |
| 11 | Tübingen | Köln |
| 12 | Leipzig | München |
| 13 | Frankfurt a.M. | Bochum |
| 14 | Kiel | Jena |
| 15 | Hannover | Kiel |
| 16 | Aachen | Frankfurt a.M. |
| 17 | Bochum | Halle (Saale) |
| 18 | Lübeck | Lübeck |
| 19 | Erlangen | Hann. Münden |
| 20 | Dresden | Mainz |
| 21 | Würzburg | Regensburg |
| 22 | Bremen | Stuttgart |
| 23 | Regensburg | Aachen |
| 24 | Augsburg | Mannheim |
| 25 | Hann. Münden | München |
| 26 | Jena | Homburg |
| 27 | Fulda | Tübingen |
| 28 | Mannheim | Köln |
| 29 | Gießen | Gießen |
| 30 | Stuttgart | Würzburg |
| 31 | Homburg | Bremen |
| 32 | Marburg | Leipzig |
| 33 | Mainz | Bonn |
| 34 |  | Marburg |
| 35 |  | Rostock |
| 36 |  | Fulda |
| 37 |  | Kaiserslautern |

**Suppl. Table 3.** Transplantation centers performing open and laparoscopic LDN according to yearly performed procedures.

| **Yearly performed LDN per center** | **<5** | **6-10** | **>10** |
| --- | --- | --- | --- |
| Laparoscopic LDN | 9 | 8 | 12 |
| Open LDN | 6 | 3 | 2 |
